# Metabolic Profiling of *Pleurotus tuoliensis* During Mycelium Physiological Maturation and Exploration on a Potential Indicator of Mycelial Maturation

**DOI:** 10.3389/fmicb.2018.03274

**Published:** 2019-01-09

**Authors:** Fang Du, Yajie Zou, Qingxiu Hu, Yunge Jing, Xiaohong Yang

**Affiliations:** Institute of Agricultural Resources and Regional Planning, Chinese Academy of Agricultural Sciences, Beijing, China

**Keywords:** *Pleurotus tuoliensis*, mycelium physiological maturation, metabolic profiling, potential indicator, *N*-carbamoyl-L-aspartate, verification

## Abstract

*Pleurotus tuoliensis* is a valuable and rare edible fungus with extremely high nutritional and medicinal value. However, the relative immaturity of *P. tuoliensis* cultivation technology leads to fluctuating yields and quality. The main difficulty in *P. tuoliensis* cultivation is estimate of mycelial maturity. There is currently no measurable indicator that clearly characterizes the physiological maturation of mycelia. The aim of this study was to identify potential indicators of physiological maturation for *P. tuoliensis* mycelia by using metabolomics approach. A metabolite profiling strategy involving gas chromatography-mass spectrometry (GC/MS) was used to analyze changes to extracellular metabolites in mycelia collected at mycelium physiological maturation period (MPMP) day 0, MPMP day 35 at 17°C and MPMP day 35 at 29°C. 72 differential metabolites (37.8% up-regulated and 62.2% down-regulated) were identified based on the selected criteria [variable important in projection (VIP) greater than 1.0 and *p* < 0.01]. Metabolic pathways enrichment analysis showed that these metabolites are involved in glycolysis, organic acid metabolism, amino acid metabolism, tricarboxylic acid cycle (TCA), sugar metabolism, nicotinate and nicotinamide metabolism, and oxidative phosphorylation. In addition, the pyrimidine synthesis pathway was significantly activated during mycelium physiological maturation of *P. tuoliensis*. The abundance of *N*-carbamoyl-L-aspartate (CA-asp), a component of this pathway, was significantly increased at MPMP day 35, which motivated us to explore its potential as an indicator for physiological maturation of mycelia. The content of CA-asp in mycelia changed in a consistent manner during physiological maturation. The feasibility of using CA-asp as an indicator for mycelial maturation requires further investigation.

## Introduction

*Pleurotus tuoliensis*, also known as *P. ferulae*, *P. eryngii* var. *tuoliensis* or *P. nebrodensis*, is a valuable and rare edible fungus (Zhao et al., 2016). It has extremely high nutritional and medicinal value. Modern pharmacological studies have shown that fungal polysaccharides contained in *P. tuoliensis* have many biological properties such as improving human immunity (Li et al., 2016), anti-tumor (Wang et al., 2014), antioxidant (Alam et al., 2012) and cholesterol-lowering activities (Alam et al., 2011). Wild *P. tuoliensis* usually grows together with the roots of *Ferula* plants in the Gobi desert, which is mainly situated in Xinjiang, China (Mou, 1987). Although large-scale commercial cultivation of *P. tuoliensis* began as early as 1997, the technology used in its production remains imperfect, leading to unstable yield and quality. It is reported that total yield of *P. tuoliensis* in 2012 reached 300 thousand tons, while in 2016 it was only 100 thousand tons (data from the China Edible Fungi Association). This is mainly ascribed to estimates on mycelium maturity. *P. tuoliensis* must undergo a mycelium physiological maturation period (MPMP) of 30–60 days, which is far longer than that of 7 days required by *P. ostreatus* and *P. eryngii* (Duan et al., 2013). This period is the process time of nutrients accumulation by mycelium metabolism. Mycelia can reach physiological maturation at the end of MPMP and the level of maturity directly affects the differentiation of primordia and the growth of fruiting bodies. Immature mycelia greatly reduce the growth rate and quality of mushrooms, while overmature mycelia have reduced viability and over-utilize nutrients in the cultivation substrate, resulting in a relative lack of nutrients during the development of fruiting body. At present, in the commercial production of *P. tuoliensis*, estimate on mycelium maturity was only based on the days of MPMP (>30 days) and mycelial growth (such as dense and thick, white in color) (Gou et al., 2003). The lack of measurable indicators that can clearly characterize the physiological maturation of *P. tuoliensis* mycelia leads to subjective evaluation of mycelium physiological maturation, resulting in fluctuations in yield and quality of *P. tuoliensis*, as well as differences in production efficiency among producers. Therefore, a clear indicator that can be used to estimate the physiological maturity of mycelia and thus provide a basis for optimal cultivation of *P. tuoliensis* is urgently needed. In addition, the uncertainty of the ambient temperature during MPMP also limited the yield of *P. tuoliensis*.

Metabolomics is a new discipline that has developed rapidly following genomics, transcriptomics and proteomics (Trivedi et al., 2017). It aims to detect the overall trajectory of endogenous metabolites in organisms or cells under specific conditions to reflect the pathological and physiological processes of organisms, and some differential metabolites detected have become potential markers that characterize the pathological and physiological states of organisms (Bracewellmilnes et al., 2017; Kaushik and Deberardinis, 2018). Metabolomics has been widely used in many fields such as disease diagnosis (Lin et al., 2017), drug development (Webhofer et al., 2011), microbial metabolism (Guo et al., 2018), and animal and plant metabolism (Pandey et al., 2016).

In recent years, metabolomics technology has been gradually applied in the field of edible fungi to study their metabolic mechanisms in response to certain environmental conditions. Zhang et al. (2008) used GC-MS-based metabolomics to study changes in volatile substances during the ripening process of *Volvariella volvacea* and *P. ostreatus*, and to evaluate the antioxidant activity of these edible fungi related to the non-volatiles and volatiles. *Agaricus bisporus* is highly susceptible to mechanical damage during storage or transportation. GC-MS technology has been used to identify metabolites that can be used as markers for *A. bisporus* injury (O’Gorman et al., 2012). Qiu et al. (2018) used GC-MS and LC-MS to analyze changes in extracellular metabolites in the mycelium of *P. ostreatus* under high temperature conditions, as well as to study the effect of exogenous addition of some significantly increased metabolites on the growth of *Trichoderma asperellum*, with the goal of determining why *P. ostreatus* was easily infected by *T*. *asperellum*. These reports above also serve as a guide to explore metabolic profiles during mycelium physiological maturation in *P. tuoliensis* by using a metabolomics approach.

In this study, GC-MS-based metabolomics was performed to study metabolic changes in mycelia collected at MPMP day 0, and day 35 under different temperatures (17 and 29°C). The primary goal of this study was to provide metabolomic information to assist in the identification of metabolites that can be used as potential indicators of mycelium physiological maturation, which could improve *P. tuoliensis* cultivation practices and thus maximize yield and quality.

## Materials and Methods

### *Pleurotus tuoliensis* Cultivation

*Pleurotus tuoliensis* (ACCC 50869) was obtained from the Agricultural Culture Collection of China. The cultivation substrate was prepared from a mixture of 40.43% cotton seed hull, 21.56% corncob, 25.16% wheat bran, 9.88% corn flour, 0.99% calcium carbonate and 1.98% lime, with moisture content of 66%. The mixed substrates were packed in mushroom culture bottles, sterilized and cooled to room temperature. Pre-cultured *P. tuoliensis* mycelia were inoculated onto the top of substrates in culture bottles. Then the inoculated bottles were kept at 25°C in the dark to encourage mycelial growth. When the substrates were fully covered with mycelia within 25 days, the culture bottles were divided into two groups, and subjected to mycelium physiological maturation period (MPMP) under a temperature of 17 and 29°C for 35 days, respectively. Thereafter, all the bottles were transferred to a mushroom house with temperature of 10–15°C and relative humidity of 90–95% to induce differentiation of primordium and development of fruiting body. Indices including cap diameter, fruiting rate, total yield and biological efficiency were recorded. Mycelia for metabolomic analysis were taken before MPMP and on day 35 of MPMP under 17 and 29°C (Table 1). Eight replicates at each time point were collected and immediately chilled in liquid nitrogen. All samples were stored at -80°C until metabolomic analysis.

**Table 1:** The information for mycelia sampling.

Index	Sampling position	Temperature	Sampling time
A	Middle part	–	MPMP, day 0
B	Middle part	17°C	MPMP, day 35
C	Middle part	29°C	MPMP, day 35


### Chemicals

All chemicals and solvents were analytical or HPLC grade. Trichloromethane was from Sinopharm Chemical Reagent Co., Ltd. (Shanghai, China). Water, methanol, pyridine, n-hexane, methoxylamine hydrochloride (97%), *N*,*O*-Bis(trimethylsilyl)trifluoroacetamide with 1% trimethylchlorosilane (BSTFA with 1% TMCS) were purchased from CNW Technologies GmbH (Düsseldorf, Germany). L-2-chlorophenylalanine was from Shanghai Hengchuang Bio-Technology Co., Ltd. (Shanghai, China).

### Sample Preparation

Mycelial extract was prepared according to the protocol reported by Qiu et al. (2018). 60 mg of accurately weighed sample were transferred to a 1.5-mL Eppendorf tube and mixed with 360 μL of cold methanol and 40 μL of 2-chloro-l-phenylalanine (0.3 mg/mL) dissolved in methanol as internal standard. Then, samples were placed at -80°C for 2 min and then grinded by a grinding machine (JXFSTPRP-24/32, Shanghai Jingxin Industrial Development Co., Ltd., Shanghai, China) at 60 Hertz (HZ) for 2 min. The mixtures were broken up by ultrasonic homogenizer (Fisher Scientific^TM^ Model 120 Sonic Dismembrator, Ottawa, Canada) at ambient temperature for 30 min. Two hundred μL of chloroform was added to the samples, and the mixtures were vortexed, with 400 μL of water added. Samples were vortexed again, then ultrasonicated at ambient temperature for 30 min. The samples were centrifuged by a high speed freezing centrifuge (TGL-16MS, Shanghai Luxiangyi Centrifuge Instrument Co., Ltd., Shanghai, China) at 12000 rpm for 10 min at 4°C. A quality control (QC) sample was prepared by mixing aliquots of all samples to form a pooled sample. Aliquots of all samples were transferred to a glass sampling vial for vacuum-drying at room temperature and 80 μL of 15 mg/mL methoxylamine hydrochloride in pyridine was subsequently added. The resultant mixture was vortexed vigorously for 2 min and incubated at 37°C for 90 min. Eighty μL of BSTFA (with 1% TMCS) and 20 μL n-hexane were added into the mixture, which was vortexed vigorously for 2 min and then derivatized at 70°C for 60 min. The samples were placed at ambient temperature for 30 min before GC-MS analysis.

### GC-MS Analysis

The derivatized samples were analyzed on an Agilent 7890B gas chromatography system coupled to an Agilent 5977A MSD system (Agilent Technologies Inc., CA, United States). A DB-5MS fused-silica capillary column (30 m × 0.25 mm × 0.25 μm, Agilent J&W Scientific, Folsom, CA, United States) was employed to separate the derivatives. Helium (>99.999%) was used as the carrier gas at a constant flow rate of 1 mL/min. The injector temperature was maintained at 260°C. Injection volume was 1 μL with 2:1 split ratio. The initial oven temperature was 60°C, ramped to 125°C at a rate of 8°C/min, to 210°C at a rate of 4°C/min, to 270°C at a rate of 5°C/min, to 305°C at a rate of 10°C/min, and finally held at 305°C for 3 min. The temperature of MS quadrupole, and ion source (electron impact) was set to 150 and 230°C, respectively. The collision energy was 70 eV. Mass data was acquired in a full-scan mode (m/z 50–500), and the solvent delay time was set to 5 min.

The QCs were injected at regular intervals (every eight samples) throughout the analytical run to provide a set of data from which repeatability can be assessed (Gika et al., 2007).

### Data Analysis

GC-MS data were exported from ChemStation (version E.02.02.1431, Agilent, United States) into common data format (CDF) and analyzed by ChromaTOF software (version 4.34, LECO, St. Joseph, MI, United States). Metabolites were qualitatived by NIST and Fiehn database. Statistic Compare component was used to process the raw data. The resulting 3-dimensional matrix containing sample information, peaks’ name, mass/retention time and peak intensities were exported as the CSV file and imported into SIMCA software (version 14.0, Umetrics, Umeå, Sweden) for multivariate statistical analysis.

Principle component analysis (PCA) and (orthogonal) partial least-squares-discriminant analysis (O)PLS-DA were performed to analyze the metabolic difference among experimental mycelia after mean centering and unit variance scaling. These metabolites, which had variable importance in the projection (VIP) value > 1 (PLS-DA) and *p* < 0.05 (two-tailed Student’s *t*-test), were identified as significant differential metabolites. Metabolic pathway enrichment analysis was performed to confirm the important pathways related to metabolic phenotype according to the KEGG website^1^.

### The Determination of *N*-Carbamoyl-L-Aspartate (CA-asp) Contents in Mycelia Collected From Different MPMP Stages

According to GC/MS differential metabolites data, CA-asp content was found to be significantly increased at MPMP day 35, which promoted us to consider whether this metabolite can be used as indicator for mycelium physiological maturity. Therefore, the mycelia were collected at MPMP days 0, 5, 10, 15, and 60, respectively, to measure CA-asp content in mycelia. Mycelial samples were dissolved in normal saline (0.15 M) in the ratio of 1: 5, and the mixture was extracted in a water bath at 37°C for 2 h. The crude enzyme solutions were obtained by filtration. CA-asp contents in crude enzymes were measured through PDAB (*p*-dimethylamino-benzaldehyde) chromogenic method described by Wang et al. (2012). 2 mL of crude enzyme solution was mixed with 2 ml of PDAB coloring solution to coloring for 15 min, and the absorbance was measured at 438 nm. CA-asp content was calculated by using a standard curve.

### Statistical Analysis

Data were analyzed using SPSS13.0 software for Windows. Differences showing *p*-value less than 0.05 were considered statistically significant.

## Results

### Effects of Different MPMP Temperatures on the Biological Properties of *P. tuoliensis*

**FIGURE 1:**
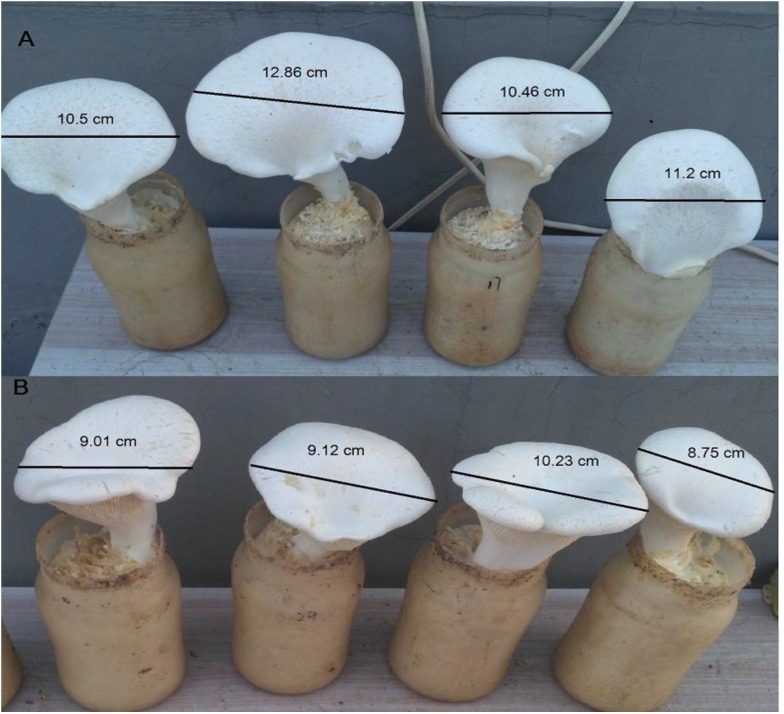
The diameter of fruiting body after a MPMP of 35 days at 17°C **(A)** and 29°C **(B)**, separately.

Cap diameter, fruiting rate, total yield and biological efficiency were recorded to evaluate the effects of different MPMP temperatures (17 and 29°C) on the development of *P. tuoliensis* (Table 2). Cap diameter is an important index for fruiting body quality, the larger the diameter, the higher the quality. As shown in Figure 1, the cap diameter of fruiting body after being subjected to an MPMP temperature of 17°C (11.95 ± 1.76 cm) was significantly larger than that of the fruiting body after subjecting to an MPMP temperature of 29°C (9.45 ± 0.89 cm). The fruiting rate and total yield after an MPMP of 35 days at 29°C (60%, 2133.60 g) were significantly lower than those measured after an MPMP of 35 days at 17°C (96%, 6057.40 g). Similarly, biological efficiency after an MPMP at 17°C was 70.35%, 51.42% higher than that after an MPMP at 29°C (46.46%).

**Table 2:** The biological properties of *P. tuoliensis* subjected to different temperatures of MPMP.

MPMP temperature (°C)	Cap diameter (cm)	Fruiting rate (%)	Total yield (g)	Biological efficiency (%)
17	11.95 ± 1.76^b∗^	96%	6057.40	70.35
29	9.45 ± 0.89^a^	60%	2133.60	46.46


*^∗^Means in each column followed by the same letters are not significantly different at *P* < 0.05.*

### Metabolic Profiles of Mycelia Into Physiological Maturity

#### GC-MS Ion Monitoring Analysis

To investigate metabolic changes in mycelia during the process of MPMP, a GC-MS approach was employed to analyze the extracellular fluid of mycelia on MPMP day 0 (Mycelia A samples), MPMP day 35 at 17°C (Mycelia B samples) and MPMP day 35 at 29°C (Mycelia C samples). Typical GC-MS total ion current (TIC) chromatograms of Mycelia A, Mycelia B, and Mycelia C samples demonstrated that instrument analysis of all samples was characterized by strong signal, high peak capacity and good reproducibility of retention time (data not shown). A total of 347 peaks were obtained, and 236 metabolites were identified among all mycelial samples. However, the biochemical changes that occurred during the process of MPMP were not apparent based on chromatograms, so a multivariate statistical analysis was employed.

#### Principal Component Analysis

**FIGURE 2:**
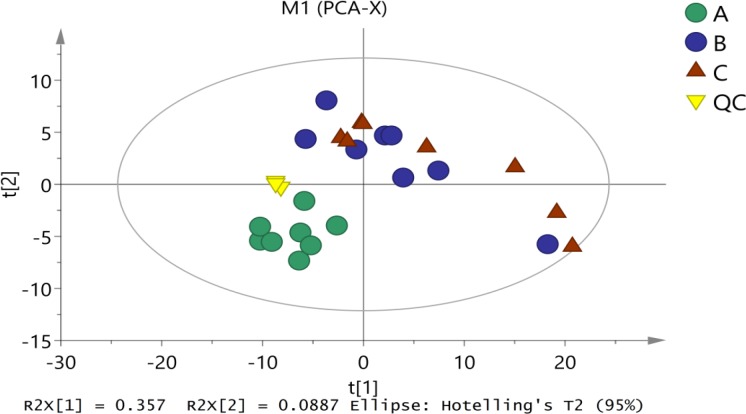
The score plots of PCA for metabolite profiles in *P. tuoliensis* mycelia. A, Mycelia A samples; B, Mycelia B samples; C, Mycelia C samples; QC, quality control sample.

First, unsupervised PCA analysis was applied to assess the overall distribution among all samples and the stability of the entire analysis process. As shown in Figure 2, four principal components of the PCA plot explain 59.1% of the total variance information. All of the QC (quality control) samples were clustered together, indicating good analytical stability and experimental reproducibility. In our PCA model, three groups of samples were well separated among the first component, indicating significant metabolic diversity among the three groups. Therein, Mycelia A samples were more concentrated and slightly away from Mycelia B and Mycelia C samples. These results are in agreement with our previous conclusion that metabolic profiles in the extracellular fluid of mycelia before and after MPMP differ greatly.

#### Partial Least-Squares Discriminant Analysis

**FIGURE 3:**
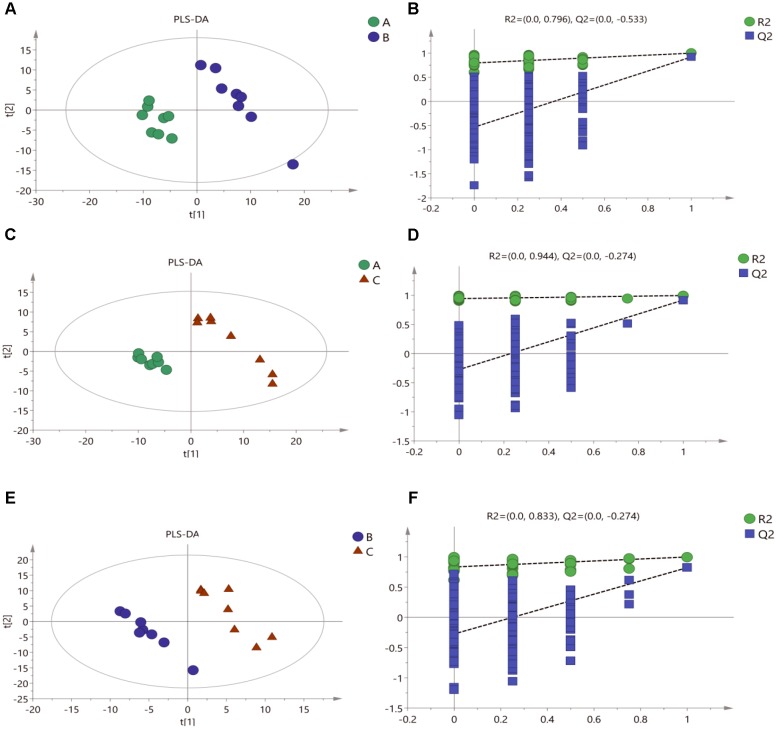
Score plots for extracellular metabolites of GC/MS data. **(A)** PLS-DA score plots from metabolite profiles for Mycelia A and B samples. **(B)** A 200 times permutation test of PLS-DA models for **(A)**. **(C)** PLS-DA score plots from metabolite profiles for Mycelia A and C samples. **(D)** A 200 times permutation test of PLS-DA models for **(C)**. **(E)** PLS-DA score plots from metabolite profiles for Mycelia B and C samples. **(F)** A 200 times permutation test of PLS-DA models for **(E)**.

To confirm the metabolic variations among three groups of mycelial samples, the group separation was further optimized using the supervised PLS-DA model. The PLS-DA pairwise comparison among Mycelia A, B, and C samples showed an obvious metabolic difference between the classes in each pairwise comparison on the 1st component (Figures 3A,C,E). The PLS-DA models were well constructed with high R2Y and Q2 values (Table 3), which indicated an excellent fit and satisfactory predictive power. A permutation test was used to evaluate possible overfitting of the PLS-DA model. In this study, a 200-time permutation test was performed. The R2-intercepts for Mycelia A, B, and C samples were 0.796, 0.944, and 0.833, whereas the Q2-intercepts were -0.533, -0.274, and -0.274, respectively (Figures 3B,D,F), indicating that the PLS-DA model showed no overfitting and was credible.

**Table 3:** Each parameter of PCA model.

No.	Type	*A*	*N*	R2X (cm)	R2Y (cum)	Q2 (cm)
All	PCA-X	4	27	0.591		0.342
B–A	PLS-DA	3	16	0.568	0.999	0.946
C–A	PLS-DA	3	16	0.579	0.997	0.954
C–B	PLS-DA	3	16	0.58	0.998	0.821


The identification of differential metabolites between pairs of samples was performed using VIP values and confirmed by the non-parametric Mann-Whitney *U* test. Among the three groups, a total of 117 differential metabolites with VIP values greater than 1.0 and *p <* 0.05 were identified, including 41 organic acids, 21 amino acids, 14 alcohols, 11 sugars, 8 amines, 5 ketones, 1 ester and 16 other metabolites. Some metabolites were rarely detected in mycelia collected at MPMP day 0 (Mycelia A sample), but showed significantly increased abundance in mycelia collected at MPMP day 35 (Mycelia B and C samples), such as CA-asp, citraconic acid, (-)-dihydrocarveol, melatonin and quindine-4-carboxylic acid. 72 differential metabolites were identified based on the criteria of VIP values greater than 1.0 and *p <* 0.01 (Table 4). The fold-changes (FC) in the abundance of each metabolite between each sample pair are also listed in Table 4.

**Table 4:** Differential metabolites among Mycelia A samples, Mycelia B samples, and Mycelia C samples identified by GC-MS.

Name	Metabolites	Fold change			Name	Metabolites	Fold change		
		B–A	C–A	C–B			B–A	C–A	C–B
Amino acid	Valine	0.56	0.36	0.65	Organic acid	Citric acid	0.28	-	3.04
	*N*-carbamoyl-L-aspartate	14.6	40.5	2.78		3-Aminoisobutyric acid	0.16	-	-
	*N*-Acetyltryptophan	0.10	0.18	-		Lauric acid	0.11	-	-
	Lysine	0.18	-	-		L-Malic acid	0.42	-	2.72
	Glycine	1.50	3.27	2.19		Stearic acid	0.55	0.54	-
	D-alanyl-D-alanine	0.61	0.51	-		6-phosphogluconic acid	0.19	-	-
	Nicotinoylglycine	1.76	2.35	-		Fumaric acid	0.58	-	1.43
	Asparagine	1.62	2.02	-		Tartaric acid	2.89	3.86	-
	*N*-Methyl-DL-alanine	1.74	2.13	-		Caprylic acid	0.11	0.30	-
	Ornithine	-	2.64	2.77		Succinic acid	0.81	0.70	-
	3-hydroxy-L-proline	-	2.29	-		Gallic acid	1.63	2.25	-
Amine	Ethanolamine	1.68	1.28	0.76		Nicotinic acid	0.17	-	-
	Nicotianamine	0.06	-	-		Arachidonic acid	1.67	2.39	-
	5-Methoxytryptamine	2.16	2.99	-		Oleic acid	0.15	0.09	-
	Adipamide	0.20	-	-		β-Glycerophosphoric acid	0.25	0.11	-
Alcohol	Myo-inositol	0.36	0.28	-		2-hydroxybutanoic acid	-	2.79	2.40
	Phytosphingosine	0.66	0.74	-		2-ketobutyric acid	-	2.77	-
	Lactitol	3.03	-	0.55		Dehydroabietic Acid	-	1.94	-
	Threitol	0.09	0.18	-		Cumic Acid	-	1.98	-
	*Cis*-1,2-Dihydronaphthalene-1,2-diol	0.05	-	14.6		Lactobionic Acid	-	0.06	-
	D-Arabitol	0.47	0.40	-		Pantothenic acid	-	0.17	-
	D-erythro-sphingosine	0.54	0.32	0.59		β-Mannosylglycerate	-	0.49	0.44
	22-Ketocholesterol	0.35	0.28	-		Pipecolinic acid	0.33	-	-
	(-)-Dihydrocarveol	-	-	3.87		4-oxo-1H-quinoline-2-carboxylic acid	0.11	-	-
	Dithioerythritol	-	2.26	-		5-aminovaleric acid lactam	1.64	2.23	-
	Xylitol	-	-	0.29		D-galacturonic acid	0.52	0.65	-
	Glycerol	0.80	0.80	-		Cytidine-monophosphate	1.36	1.95	-
Sugar	Melezitose	0.13	-	-		Dibenzofuran	1.69	2.17	-
	Xylose	-	0.04	-		1-Methylhydantoin	-	1.98	-
	Sedoheptulose	0.62	-	-		3-Hydroxypyridine	0.19	-	-
	Ribose	0.07	0.14	-	Others	5,6-dihydrouracil	0.62	-	1.77
	D-Talose	0.44	0.18	0.40		*p*-benzoquinone	-	2.21	-
	D-Altrose	0.18	0.09	-		6-Methylmerceptopurine	1.59	1.94	-
	Maltotriose	0.57	0.18	0.31		Squalene	1.74	2.14	-
	Ribose-5-phosphate	0.20	0.08	-		2,3-Dihydroxypyridine	0.14	-	10.8


#### Hierarchical Cluster Analysis

**FIGURE 4:**
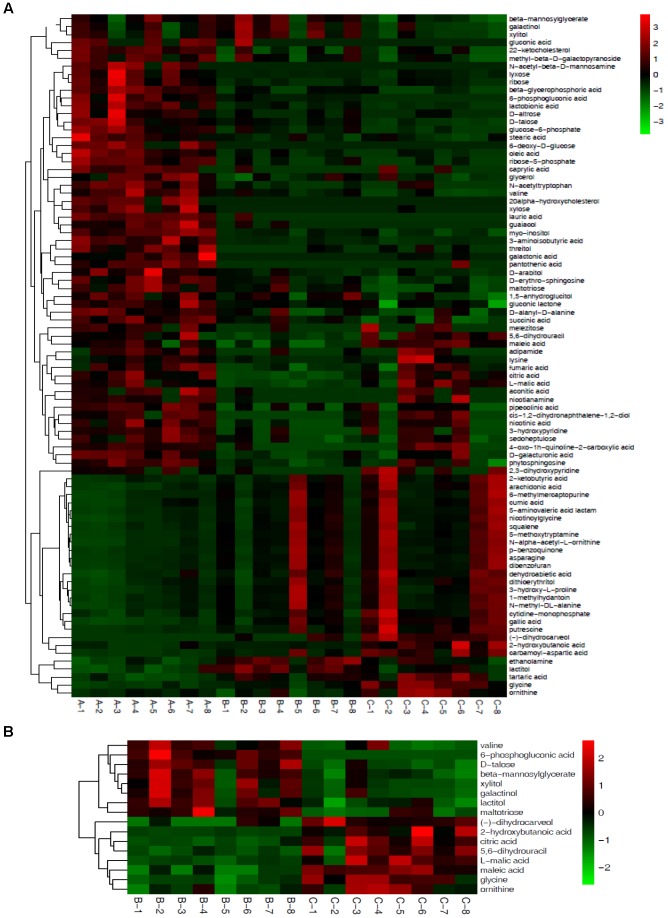
Heat maps of differential metabolites among different mycelia samples. Each mycelium sample is visualized in a single column and each metabolite is represented by a single row. Green colors indicate lower metabolite concentration, while red colors show enhanced metabolite levels (see color scale). See also Table 4. **(A)** Heat map visualization of differential metabolites among Mycelia A samples, Mycelia B samples and Mycelia C samples. **(B)** Heat map visualization of differential metabolites between Mycelia B samples and Mycelia C samples.

**FIGURE 5:**
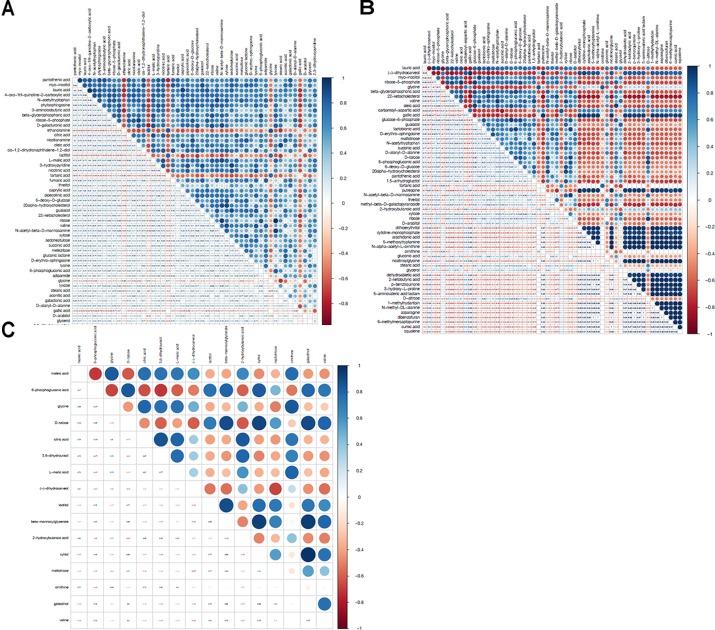
The correlation analysis between differential metabolites in Mycelia B samples and Mycelia A samples **(A)**, Mycelia C samples and Mycelia A samples **(B)**, and Mycelia C samples and Mycelia B samples **(C)**. Blue color represents positive correlation, while red color represents negative correlation (see color scale). The value which is larger demonstrates the greater correlation between the two metabolites.

Based on the 72 annotated metabolites, clear dynamic changes were observed in the heat map analysis among Mycelia A, B, and C samples (Figure 4A). The upper part of Figure 4A shows metabolites with significantly decreased abundance in Mycelia B and Mycelia C samples in comparison with Mycelia A samples, while the lower part of Figure 4A indicates metabolites with significantly up-regulated in Mycelia B and Mycelia C samples in comparison with Mycelia A samples, such as CA-asp, glycine, lactitol, tartaric acid, lactam, squalene, dibenzofuran, 6-methylmercaptopurine, 5-methoxytryptamine, ethanolamine, asparagine, gallic acid, and nicotinoylglycine. Figure 4B shows differential metabolites in Mycelia C samples in comparison with Mycelia B samples. Metabolites with significantly increased abundance in Mycelia B samples in comparison with Mycelia C samples include valine, 6-phosphogluconic acid, D-talose, β-mannosylglycerate, xylitol, galactinol, lactitol, and maltotriose. Whereas, the abundance of (-)-dihydrocarveol, 2-hydroxybutanoic acid, citric acid, 5,6-dihydrouracil, L-malic acid, maleic acid, glycine, and ornithine was significantly decreased. Figure 5 shows the correlation analysis of differential metabolites between Mycelia B samples and Mycelia A samples (A), between Mycelia C samples and Mycelia A samples (B), and between Mycelia C samples and Mycelia B samples (C). The value which is larger demonstrates the greater correlation between the two metabolites.

#### Metabolic Pathway Analysis

**FIGURE 6:**
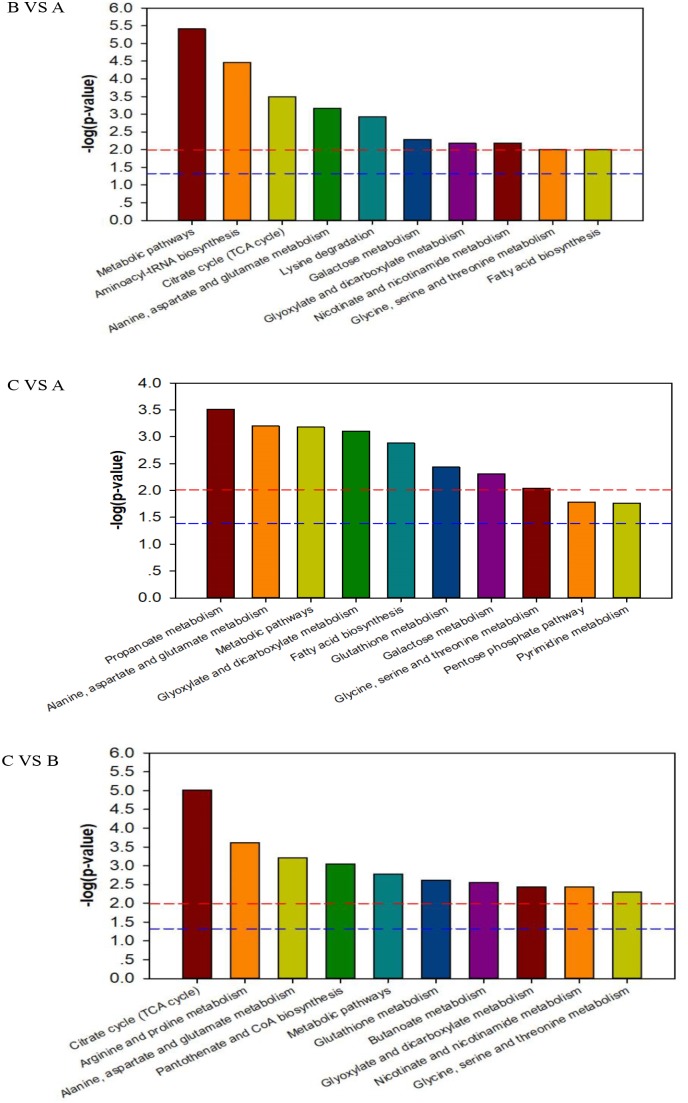
The KEGG pathway enrichment analysis of differential metabolites between Mycelia B samples versus Mycelia A samples (B vs. A), Mycelia C samples versus Mycelia A samples (C vs. A), and Mycelia C samples versus Mycelia B samples (C vs. B). Each comparison mycelia only shows top 10 enrichments pathways of differential metabolites. P-value was calculated using Fisher’s exact test.

**FIGURE 7:**
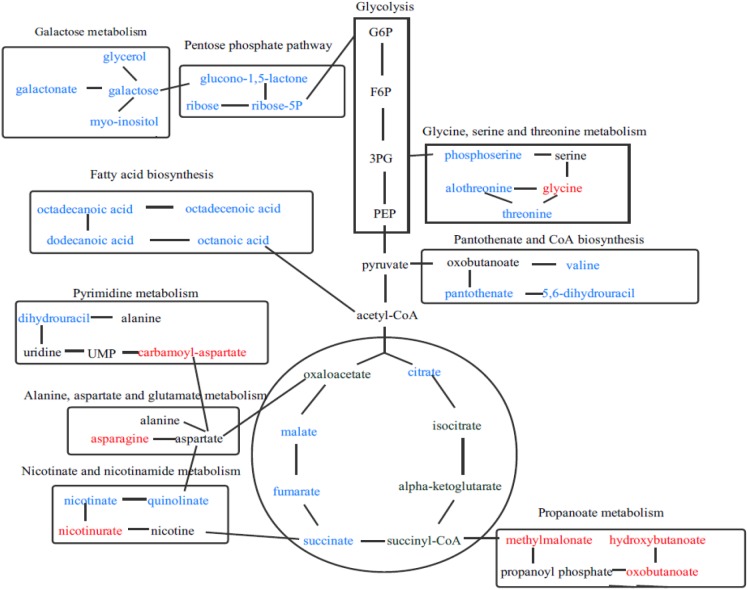
The relationship among some significantly changed pathways and differential metabolites involved in each metabolic pathway. Metabolites in red indicate lower concentration, while metabolites in blue show enhanced levels.

**FIGURE 8:**
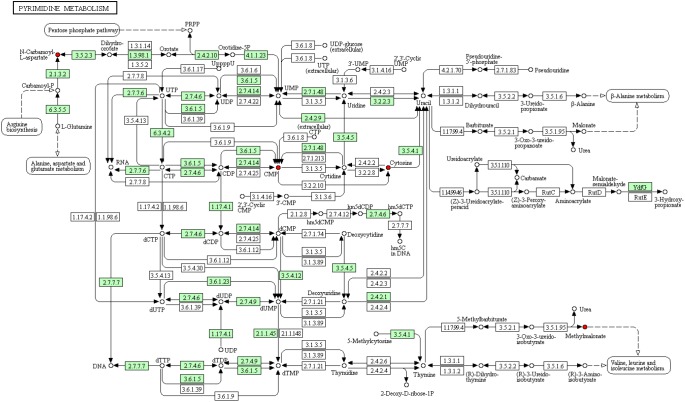
Pyrimidine metabolic pathways in mycelia after physiological maturation. Red cycle indicates up-regulated metabolite, arrow indicates reacted direction. Small green box represents the enzyme specific to the species. Dotted line indicates relationships with other metabolic pathways. Big cycle box represents other metabolic pathways.

To deeply understand the differences in the metabolic networks among three groups of samples, 72 differential metabolites identified were submitted to the KEGG website for metabolic pathway enrichment analysis. Figure 6 shows enriched pathways between Mycelia B and Mycelia A samples, Mycelia C and Mycelia A samples, and Mycelia C and Mycelia B samples, including five primary metabolic pathways [glycolysis, organic acid metabolism, amino acid metabolism, tricarboxylic acid cycle (TCA) and sugar metabolism], and some secondary metabolic pathways (nicotinate and nicotinamide metabolism and oxidative phosphorylation). Some significantly changed pathways and differential metabolites involved in each metabolic pathway are displayed in Figure 7. Figure 8 solely displays the whole pyrimidine metabolic pathway which was significantly activated on day 35 of MPMP.

#### CA-asp Content in Mycelial Samples Collected at Different MPMP Stages

**FIGURE 9:**
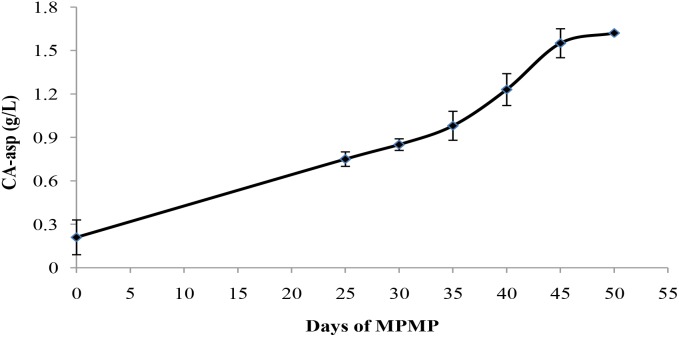
*N*-carbamoyl-L-aspartate (CA-asp) content in mycelia collected at different MPMP stages.

To evaluate the feasibility of CA-asp as an indicator for mycelium physiological maturation, CA-asp content was measured in mycelia collected at different MPMP stages. As shown in Figure 9, CA-asp content gradually increased during the MPMP. Its content was only 0.21 g/L when *P. tuoliensis* mycelia had just entered physiological maturation stage (MPMP day 0), and remarkably ascended to 1.55 g/L at MPMP day 45, after which it slowly increased and reached 1.60 g/L at MPMP day 50, but this latter change was not significant in comparison with CA-asp content at day 45.

## Discussion

Mycelium physiological maturation period is an extremely important stage before primordia formation. Mycelia reach physiological maturity during the process of MPMP, and their level of maturity is positively correlated with their fruiting rate. In industrial production, thick and white mycelia and a compact cultivation bag are usually used as appearance indicators for judging the physiological maturity of mycelia, but no measurable signals have been found. In addition, we have confirmed that *P. tuoliensis* MPMP lasting 35 days at 17°C resulted in larger cap diameters, higher fruiting rates, increased yields and improved biological efficiency, in comparison with an MPMP of 35 days at 29°C, but the reason lower temperatures during MPMP facilitate fruiting body development remains unclear.

Some metabolites in mycelia change greatly during physiological maturation. Thus they may be important signals of entry into primordia differentiation stage. Metabolomics provides an effective approach for studying the metabolic profiles of mycelia before and after MPMP, as well as during temperature stress (Zhang et al., 2014). In our study, GC-MS-based metabolomics was performed to screen for significantly altered metabolites in mycelia at MPMP day 0 (Mycelia A samples), MPMP day 35 at 17°C (Mycelia B samples) and MPMP day 35 at 29°C (Mycelia C samples), with the goal of identifying potential biomarkers for mycelial maturation. Multivariate PCA and OPLS-DA analyses, which could confirm the intrinsic variation of metabolites and the stability of the entire analysis process (Song et al., 2013; Brusco, 2014), showed that there were changes in the composition and concentrations of extracellular metabolites among three tested groups of *P. tuoliensis* mycelia, which indicated that metabolic profiles were influenced by the growth of the mycelia as well as by temperature stress.

Using statistical analysis and VIP values obtained from the OPLS-DA analysis, 72 differential metabolites were found to be greatly altered among three tested groups of mycelium samples (*p* < 0.01 and VIP > 1.0). A heat map (Figure 4A) showed that the abundance of 37.8% of identified extracellular metabolites was significantly increased, while that of 62.8% of identified extracellular metabolites decreased at MPMP day 35. The results of metabolic pathway enrichment analysis indicated that many of the identified metabolites were involved in sugar metabolism, amino acid metabolism, and tricarboxylic acid (TCA) cycle.

Tricarboxylic acid cycle is a key metabolic pathway that unifies protein, fat and carbohydrate metabolism (Wahyuni et al., 2013). The reactions of TCA cycle are carried out by eight enzymes that completely oxidize acetyl-CoA into two molecules of carbon dioxide. Through catabolism of proteins, fats and sugars, acetate is produced in the form of acetyl-CoA, which enters the citric acid cycle (Vagelos et al., 1963). A decrease of citrate, succinate, fumarate, malate, and ribose in physiologically mature mycelia suggested that TCA cycle was depressed and mycelium metabolism was slowed down. We speculate that the slowing of metabolism is to prevent mycelia from excessive decomposition of cultivation substrate, and thus avoid insufficient nutrients during the formation of primordia and fruiting bodies.

The concentrations of all measured sugars were decreased in mature mycelia. This phenomenon was in accordance with growth profiles of fungal mycelia. Basidiomycetes, such as the oyster mushroom, king oyster mushroom, winter mushroom, and shiitake, can enzymatically degrade cultivation substrates containing lignin, hemicellulose, and cellulose into low molecular weight sugars, which are then absorbed by fungal hyphae to support growth and maturation in a process called nutritive absorption (Lim et al., 2012, 2013; Chen et al., 2016). In the late stage of mycelial maturation, the slowing of metabolism causes a decrease in the levels of extracellular enzymes, so the abundance of degradation products is correspondingly reduced. In our study, an extremely low concentration of ribose, a key intermediate of pentose phosphate pathway, was observed in mature mycelia, suggesting that this pathway was also depressed. The pentose phosphate pathway is a biochemical pathway that functions in parallel with glycolysis and generates nicotinamide adenine dinucleotide phosphate (NADPH) and pentoses (5-carbon sugars) and involves oxidation of glucose. Therefore, its primary role is anabolic rather than catabolic (Kruger and Von, 2003; Keller et al., 2014). The pentose phosphate pathway is a major source of reductants for biosynthetic processes such as fatty-acid synthesis and assimilation of inorganic nitrogen (Emes and Neuhaus, 1995). In our study, the abundance of octanoic acid, dodecanoic acid, octadecanoic acid, and octadecenoic acid in fatty acid synthesis pathway were sharply decreased at MPMP day 35. The reductant used for fatty acid synthesis originated mainly from pentose phosphate pathway, which was found to be depressed. Therefore down-regulation of these fatty acids was in good agreement with that of pentose phosphate pathway intermediates.

Most amino acids identified in this study were evidently abundant in mycelia at MPMP day 35, suggesting that amino acid metabolism was essential during mycelial maturation. The abundance of glycine, a glucogenic amino acid, which could be converted into glucose via gluconeogenesis (Brosnan, 2003), was significantly elevated at MPMP day 35 (*p* < 0.05). Aspartate is mainly from oxaloacetate, which is involved in TCA cycle (Scandiani et al., 2015). The abundance of aspartate showed insignificant changes (*p* < 0.05) after maturation. But aspartate-derived amino acids (asparagine and CA-asp) accumulated significantly in mature mycelia (1.62- and 2.02-fold for asparagine at 17 and 29°C, respectively, and 14.6- and 40.5-fold increases for CA-asp at 17 and 29°C, respectively). The increases in the abundance of asparagine and CA-asp were attributed to active participation of other metabolic pathways, and not to the presence of alanine, aspartate, and glutamate metabolism in which aspartate is involved.

Our observations indicate that pyrimidine synthesis, which is related to CA-asp, was significantly activated, suggesting that physiological maturation of mycelia increased the demand for nucleotides for the rapid synthesis of cellular materials, which was fulfilled by *de novo* synthesis and/or nucleotide salvage of pyrimidines. Figure 8 shows the entire pyrimidine metabolic pathway in mycelia after physiological maturation (intermediates with significantly increased abundance are shown in red). Mature mycelia showed elevated concentrations of nucleotides, including cytidine monophosphate (CMP) and cytosine, which were indicative of the high demand for rapid nucleotide synthesis during physiological maturation. Methylmalonate is a degradation product that is catalyzed by methylbarbiturate and is associated with valine, leucine and isoleucine metabolism. The increase in the abundance of methylmalonate in mature mycelia might mean that the corresponding metabolic network was more active than that in immature mycelia. Moreover, extracellular nucleotides stimulate mycelial survival via purinergic signaling (Schneider et al., 2015).

As stated above, MPMP temperature influences the yield and quality of fruiting bodies, which is also reflected in differences in the abundance of metabolites. The concentrations of citric acid, maleic acid and L-malic acid in Mycelia C samples (at 29°C) were significantly higher than those in Mycelia B samples (at 17°C) at MPMP day 35 (Figure 4B). These differential metabolites were involved in TCA cycle, which is related to energy metabolism. Therefore, the metabolism of Mycelia C samples was likely more active than that of Mycelia C samples, indicating that Mycelia C samples consumed more cultivation substrate, which led to insufficient concentrations of nutrients for the formation of primordia and fruiting bodies. This difference also explains why mycelia that were exposed to low temperatures during physiological maturation may have larger cap diameters, higher fruiting rates, increased yield and improved biological efficiency.

Among the identified metabolites, CA-asp attracted our attention because of high fold-change difference in its abundance in Mycelia B and Mycelia C samples in comparison with its abundance in Mycelia A samples. Interestingly, almost no CA-asp was detected in mycelia collected at day 0 of the MPMP, but its content was extremely high in physiologically mature mycelia. We deduced that CA-asp may be used as a potential indicator to identify whether mycelia have reached physiological maturity. Therefore, we determined the content of CA-asp in mycelia at different MPMP stages, which revealed that CA-asp content continued to rise until day 50 of the MPMP. Therefore, in our study, the content of CA-asp in mycelia showed a regular change during physiological maturation. However, the utility of CA-asp content as an indicator of the physiological maturation of mycelia was not determined, and this question merits additional fruiting experiments.

## Conclusion

Our cultivation results demonstrated that a lower MPMP temperature (17°C) is beneficial for the growth and development of *P. tuoliensis*. A GC-MS-based metabolic approach was employed to compare the abundance of metabolites in mycelia sampled at MPMP day 0, MPMP day 35 (17°C) and MPMP day 35 (29°C). 236 differential metabolites were identified. For the purpose of analysis, 72 differential metabolites (37.8% up-regulated and 62.2% down-regulated) were selected based on the criteria of VIP greater than 1 and *p* < 0.01. These metabolites are mainly involved in glycolysis, organic acid metabolism, amino acid metabolism, TCA cycle, sugar metabolism, nicotinate and nicotinamide metabolism, and oxidative phosphorylation. The pyrimidine metabolic pathway may be related to the physiological maturation of mycelia, and metabolite CA-asp which participated in this pathway can be a potential indicator for mycelial maturation, because its abundance was significantly enhanced at MPMP day 35. Subsequently, we proved that CA-asp content in mycelia collected at different MPMP stages showed a regular change, which indicated the feasibility of CA-asp used as indicator for mycelial maturation, but whether this indicator can be applied for the commercial production of *P. tuoliensis* requires the verification of large-scale fruiting experiences.

## Author Contributions

FD, YZ, YJ, and XY conceived, designed, and performed the experiments, analyzed the data, and wrote and revised the manuscript. QH conceived and designed the experiments.

## Conflict of Interest Statement

The authors declare that the research was conducted in the absence of any commercial or financial relationships that could be construed as a potential conflict of interest.

## Footnotes

1 www.genome.jp/kegg/
